# Energy enhanced tissue texture in spectral computed tomography for lesion classification

**DOI:** 10.1186/s42492-019-0028-3

**Published:** 2019-11-18

**Authors:** Yongfeng Gao, Yongyi Shi, Weiguo Cao, Shu Zhang, Zhengrong Liang

**Affiliations:** 1 Department of Radiology, Stony Brook University, Stony Brook, NY 11794 USA; 2 Institute of Image Processing and Pattern Recognition, Xi’an Jiaotong University, Xi’an, 710049 Shanxi China; 3 Departments of Radiology, Biomedical Engineering, Computer Science, and Electrical Engineering, Stony Brook University, Stony Brook, NY 11794 USA

**Keywords:** Tissue texture, Spectral computed tomography, Lesion classification, Machine learning, Bayesian reconstruction

## Abstract

Tissue texture reflects the spatial distribution of contrasts of image voxel gray levels, i.e., the tissue heterogeneity, and has been recognized as important biomarkers in various clinical tasks. Spectral computed tomography (CT) is believed to be able to enrich tissue texture by providing different voxel contrast images using different X-ray energies. Therefore, this paper aims to address two related issues for clinical usage of spectral CT, especially the photon counting CT (PCCT): (1) texture enhancement by spectral CT image reconstruction, and (2) spectral energy enriched tissue texture for improved lesion classification. For issue (1), we recently proposed a tissue-specific texture prior in addition to low rank prior for the individual energy-channel low-count image reconstruction problems in PCCT under the Bayesian theory. Reconstruction results showed the proposed method outperforms existing methods of total variation (TV), low-rank TV and tensor dictionary learning in terms of not only preserving texture features but also suppressing image noise. For issue (2), this paper will investigate three models to incorporate the enriched texture by PCCT in accordance with three types of inputs: one is the spectral images, another is the co-occurrence matrices (CMs) extracted from the spectral images, and the third one is the Haralick features (HF) extracted from the CMs. Studies were performed on simulated photon counting data by introducing attenuation-energy response curve to the traditional CT images from energy integration detectors. Classification results showed the spectral CT enriched texture model can improve the area under the receiver operating characteristic curve (AUC) score by 7.3%, 0.42% and 3.0% for the spectral images, CMs and HFs respectively on the five-energy spectral data over the original single energy data only. The CM- and HF-inputs can achieve the best AUC of 0.934 and 0.927. This texture themed study shows the insight that incorporating clinical important prior information, e.g., tissue texture in this paper, into the medical imaging, such as the upstream image reconstruction, the downstream diagnosis, and so on, can benefit the clinical tasks.

## Introduction

Tissue texture reflects the spatial distribution of contrast of image voxel gray levels across the field of view [1, 2], which is an effective descriptor of tissue heterogeneity. It has been recognized as an important biomarker in various clinical tasks, such as tumor type classification, tumor treatment response evaluation [3, 4]. Tissue texture can be enhanced by spectral computed tomography (CT) because spectral CT can provide a set of CT images with different voxel contrast using different X-ray energy. For example, by recent photon counting spectral CT (PCCT) technology, typically five energy channel images can be obtained [5]. To make full use of energy enriched texture by spectral CT for clinical tasks, this paper aims to address two related issues: (1) texture enhancement in spectral CT image reconstruction; and (2) spectral energy enriched tissue texture for lesion classification.

Reconstructing high quality spectral CT images is essential, where the tissue texture should be preserved because it contains high clinical importance. PCCT image reconstruction is subjected to photon starving problems [6, 7]. Photon counting technology enables detector to discriminate photons in certain energy range. If the total photon counts are the same with the traditional CT, the low photon counts will be suffered in each individual energy channel. Model based iterative reconstruction method under Bayesian law has been widely used to deal with such a low photon counts problem [8, 9], where the *prior* term enables us to incorporate our human prior knowledge or constraints for the purposes of noise reduction, edge sharpness preservation, and so on. To enhance the tissue texture information for PCCT, we recently proposed a tissue-specific texture prior (TP) [10–13] in addition to low rank (LR) prior*,* denoted as low rank texture prior (LRTP) algorithm to reconstruct images of multiple energy channels as a tensor [14]. Promising results were reconstructed for lung imaging.

Using the spectral enriched tissue texture for lesion classification will be another important issue once we would have obtained high quality spectral CT images. To our best knowledge, only a few studies [15–19] are reported using spectral CT enriched data for lesion classification. In ref. [16], the authors use the iodine concentrations in the lesions and lymph nodes measured from the material-decomposition images for the gastric cancer staging diagnosis. Similar studies are also performed in refs. [17–19]. In ref. [15], the authors use the virtual monochromatic images (VMI) obtained from dual energy CT for benign parotid tumors classification, where six texture features such as mean, standard deviation, entropy, etc., are used. It is realized that extracting enriched texture information from spectral CT for the classification task is challenging. As the artificial intelligence (AI) being developed, the machine learning, especially deep learning-based method has been applied successfully in the medical imaging field [20–23]. To benefit from the AI, the simplest way is to throw the spectral CT images directly to the deep learning model and ask machine to extract high level features. However, this way may require huge data for training and sometimes impossible in medical field. Another way is to incorporate our human knowledge, e.g., the tissue texture in the classification model. Hence, this paper investigates three models in terms of three type inputs: one is the spectral images, another is the co-occurrence matrices (CMs) extracted from spectral images, and the third one is the Haralick features (HF) extracted from CMs. The second and third models basically use the texture descriptor as inputs. We will describe three models detailed in the method section. This investigation will not only validate whether the enriched texture by spectral CT will benefit the classification task but also provide us guidelines for how to use the enriched texture.

By addressing the two important issues above, this paper shows the insight of incorporating the clinically important prior information, e.g., tissue texture in this paper, to increase the PCCT potential by the upstream image reconstruction, improve the outcome of the downstream computer-aided diagnosis, and so on.

## Methods

### LRTP for spectral CT image reconstruction

As mentioned in introduction, the image reconstruction for each individual energy channel is a low photon counts problem, which can be relieved by the Bayesian type image reconstruction method. We recently proposed one tissue-specific TP in addition to LR prior under the Bayesian law to enhance the tissue texture in the image reconstruction. Firstly, to make use of the synergy among the individual energy channel, the inter-channel correlation is modeled by low-rank representation technique. Secondly, the inner-channel spatial texture of the target corrupted images is characterized by TP, which is convex and can well preserve the edges and texture features. The proposed LRTP method integrates the inter-channel correlation and the inner-channel spatial texture into a unified Bayesian reconstruction framework. The overall cost function can be expressed as:

1 $$ \arg {\min}_{\upchi}\parallel \mathcal{A}\left(\upchi \right)-\mathcal{Y}{\parallel}_2^2+\lambda {\parallel}_{tr}+\beta R\left(\upchi \right) $$

The TP prior is inspired by our previous studies in traditional CT image reconstruction [16–20], which can be formulated as:

2 $$ R\left(\upchi \right)=\sum \limits_k\sum \limits_{r=1}^R\sum \limits_{j\in \mathrm{Region}(r)}\sum \limits_{m\in {\Omega}_j}{w}_{jmk}^{FD}{\left({x}_{jk}-{x}_{mk}\right)}^2 $$

where index *k* denotes the energy channel, index *j* runs over all the pixels in each channel image, Ω_*j*_ represents a small fixed neighborhood window of the *j-th* pixel in the channel image, ${w}_{jmk}^{FD}$ is the weighting coefficient which represent the correlation between pixel *m* and pixel *j* in *k-th* channel predicting from full-dose images, *r* specifies the tissue type, *R* is the total number tissue regions.

**Fig. 1:**
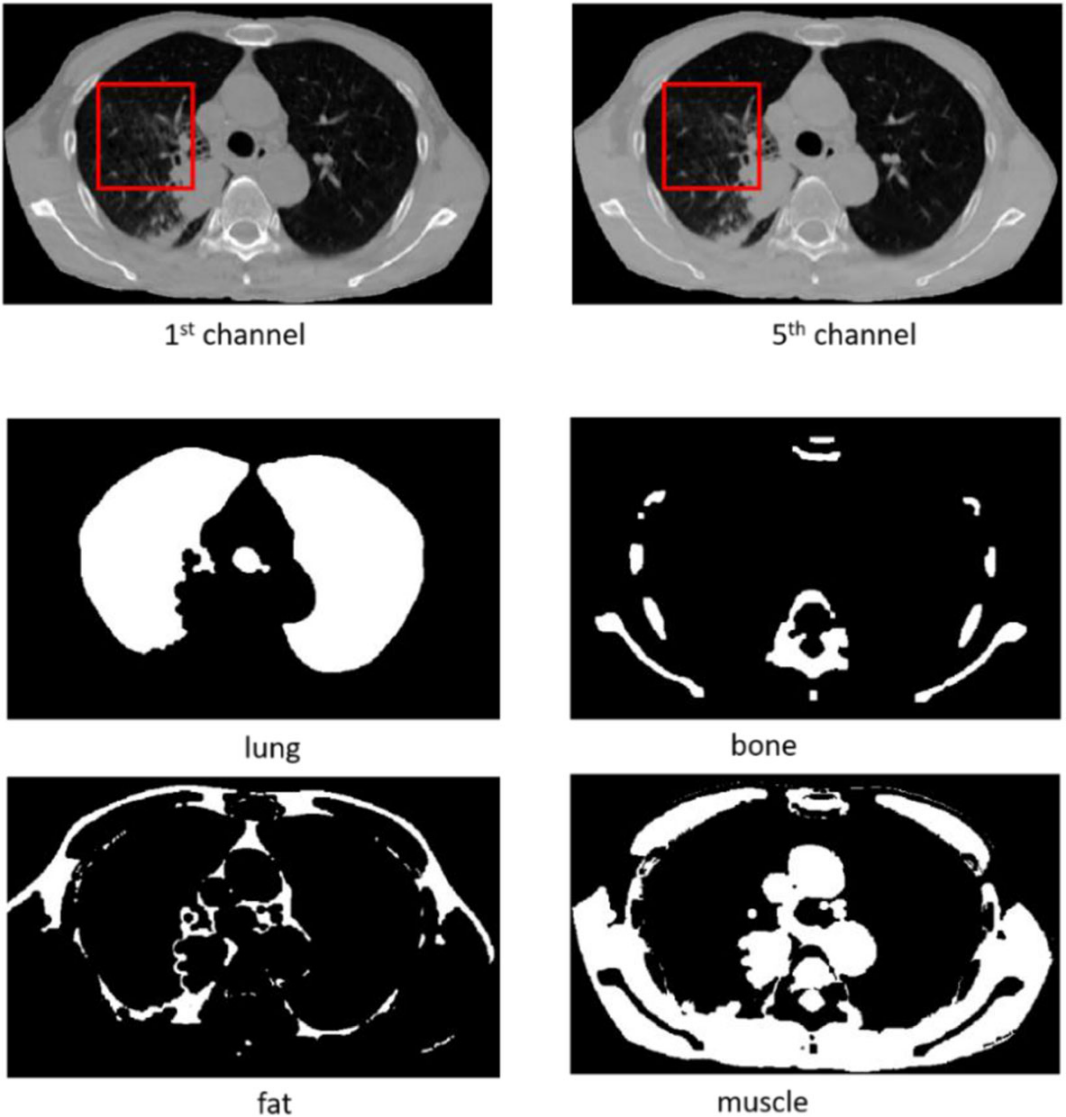
Full dose computed tomography images and their segmented tissue masks. Images of first and last (5th) energy channel. Tissue masks of lung, bone, fat and muscle

**Fig. 2:**
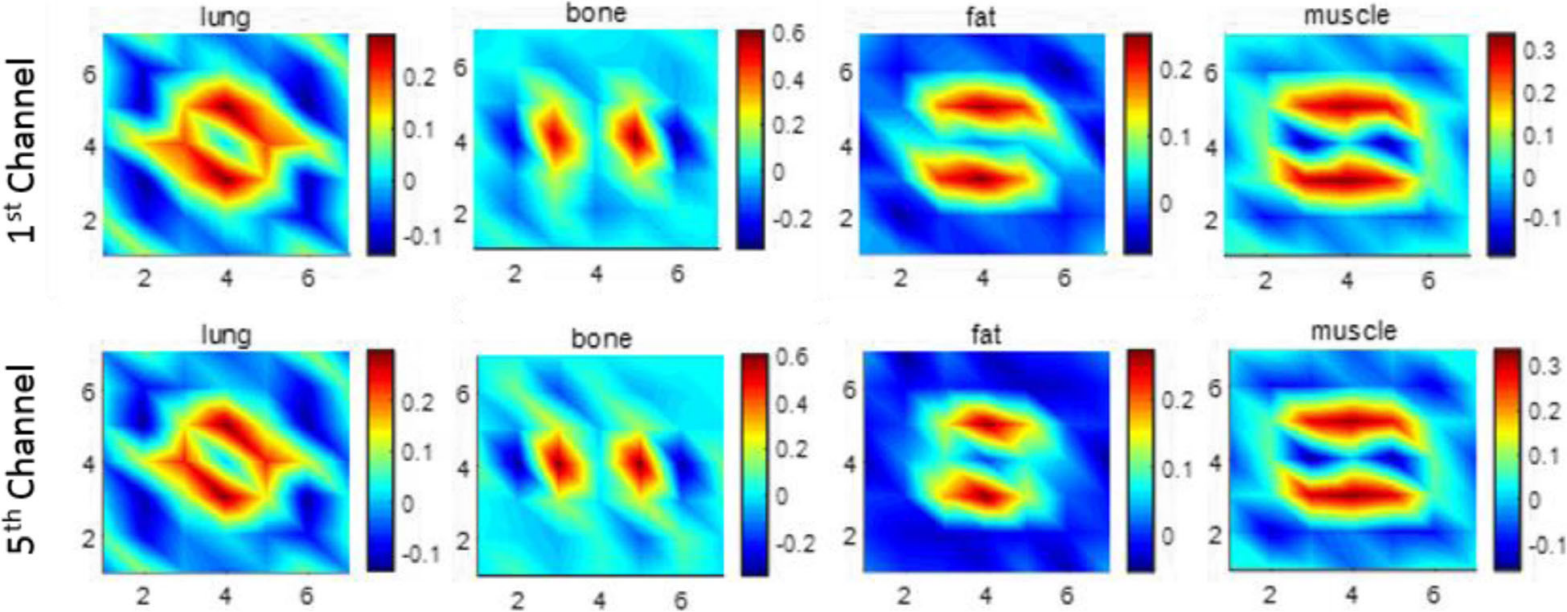
The predicted markov random field coefficients for the four tissue regions of lung, bone, fat and muscle. The top panel is for the first channel. The bottom panel is for the last channel. From left to right, markov random field weights of lung, bone fat and muscle are shown respectively

In this study, the tissue type number is setting as *R* = 4 to represent lung, fat, muscle and bone for chest CT imaging. Reconstructed full dose CT (FDCT) images were segmented using our previously reported vector quantization algorithm [24]. Figure 1 shows one example of reconstructed FDCT images of first and last (the 5th) energy channel. The segmented tissue masks are also shown in Fig. 1. Given the segmented masks of the four tissues, we apply Eq. (2) to calculate the corresponding markov random field (MRF) coefficients, of which the window size is setting to 7 × 7. Figure 2 demonstrates the predicted MRF coefficients set of the four tissue regions in the first (top panel) and last channel (bottom panel). We can observe that MRF coefficients in the same tissue region among different channels have strong similarity. It agrees with our hypothesis that strong correlation exists among energy channels, which motivates us to use the LR prior in the image reconstruction. However, there still have some differences. For example, in the muscle plot of Fig. 2, the red region of the first channel is larger than that of the last channel. This is also expected because the attenuation coefficient differs under different X-ray energy.

Since this paper focus on texture themed discussion, details of the proposed LPTR is not included and can be found in ref. [14]. Implementation of LRTP is summarized in Table 1.

**Table 1:** Workflow for the proposed low rank texture prior algorithm

Texture learning	
Learning the tissue-specific texture from corresponding full-dose image.	
Image reconstruction	
Initialize $\mathcal{X}$ by algebraic reconstruction technique [25];	
Set parameters *λ*, *μ*, *β*.	
While stop criterion is not met:	
Setp1: ${\mathcal{X}}^{t+1}={\mathrm{argmin}}_{\mathcal{X}}{\left\Vert \mathcal{AX}-\mathcal{Y}\right\Vert}_2^2+\beta R\left(\mathcal{X}\right)+\mu {\left\Vert {\mathcal{D}}^t-\mathcal{X}-{\mathcal{V}}^t\right\Vert}_2^2$;	
Step2: ${\mathcal{D}}^{t+1}={\mathrm{argmin}}_{\mathcal{D}}\lambda {\left|\mathcal{D}\right|}_{\ast }+\mu {\left\Vert \mathcal{D}-{\mathcal{X}}^{t+1}-{\mathcal{V}}^t\right\Vert}_2^2$;	
Step3: ${\mathcal{V}}^{t+1}={\mathcal{V}}^t+{\mathcal{X}}^{t+1}-{\mathcal{D}}^{t+1}$;	
End until the stop criterion is satisfied.	

### Enriched texture classification models

**Fig. 3:**
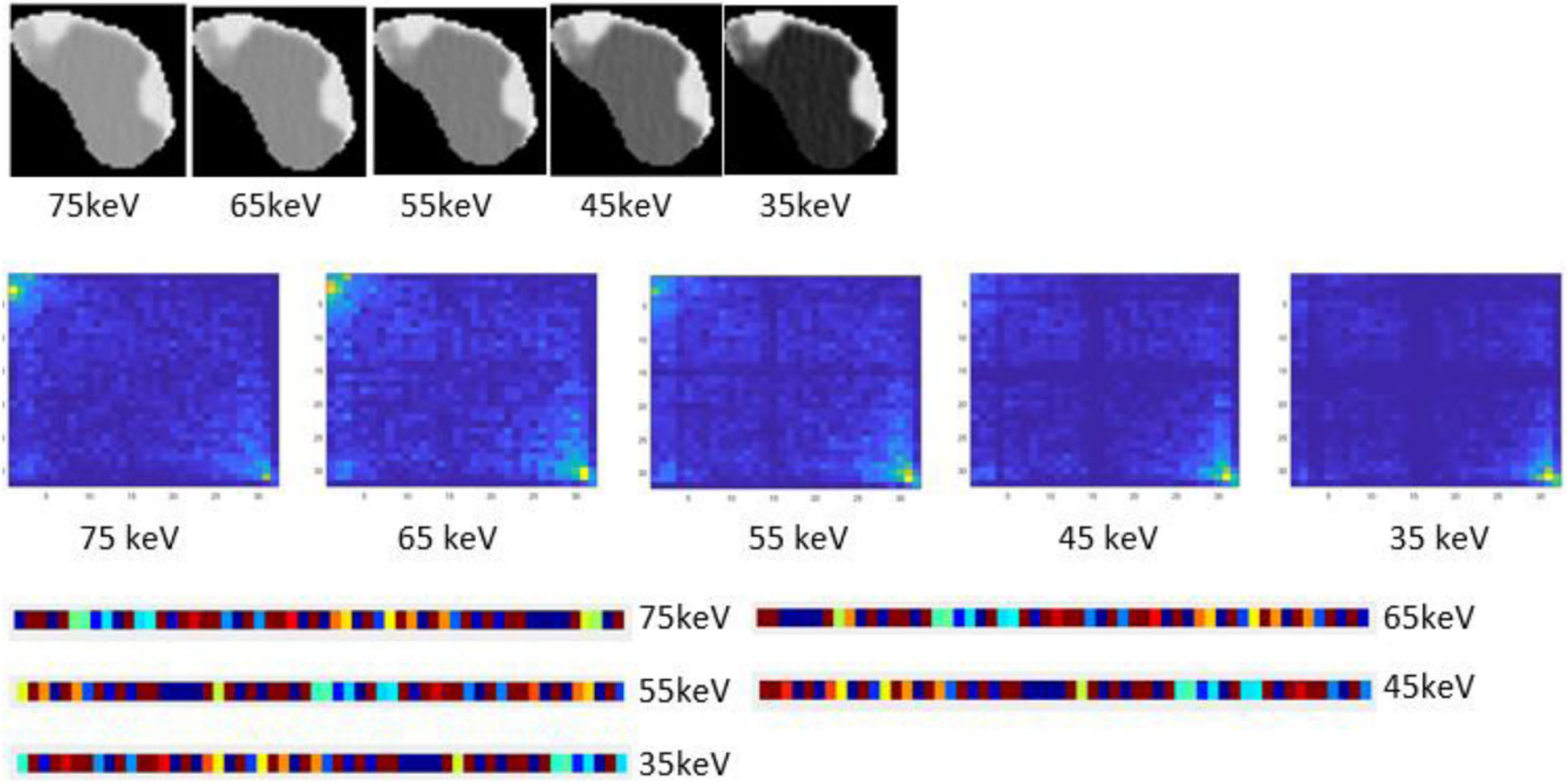
An example of one polyp slice at different energy channel and the corresponding extracted texture features. The first row is the raw computed tomography image; the second row is the co-occurrence matrices (CMs) extracted from the spectral images; the third row is the Haralick features extracted from the CMs

As introduced above, spectral CT can enrich tissue texture by providing set of CT images with different contrast. The first row in Fig. 3 showed an example set of virtual monochromatic CT images with X-ray energy from 35, 45, 55, 65 and 75 keV respectively. It is clearly observed that the pixel contrast varies significantly under different energy. The five-energy spectral CT much enriched the texture and is expected to provide more information. However, it remains challenging and pioneering using the enriched texture for clinical tasks like predict the tumor type, cancer stage, etc.

In this paper, we investigated three classification models in terms of three type inputs: one is the spectral images, one is the CMs extracted from the spectral images, and the third one is the HF extracted from the CMs. As we discussed earlier, the spectral CT images have already enriched the tissue textures. The simplest way is to throw the set of images directly into a machine learning model and ask the machine to extract the effective features from each energy image and then fuse these features to generate higher level features for the final prediction. However, this way may require huge training data samples, which is impossible in medical field at present or near future. Thus, we can incorporate our prior knowledge of the texture into the model.

**Fig. 4:**
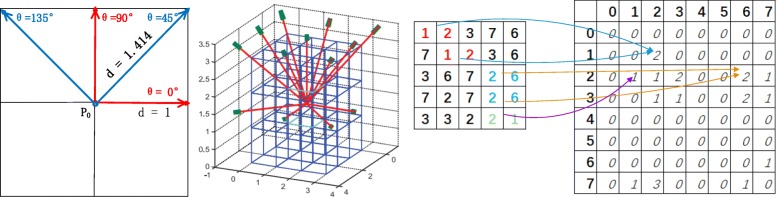
Illustration of grey level co-occurrence matrices of 2 dimensional /3 dimensional images. **Left pannel**: 2 dimensional grey level co-occurrence matrices (GLCM) calculation; **Middle pannel**: 3 dimensional GLCM calculation; **Right pannel**: A GLCM example when angle(θ) = 0°, displacement(d) = 1

Since the texture reflects the voxel grey level distribution [2], we can quantify this distribution through the mathematic process. Tremendous efforts have been devoted developing texture descriptors [26–28]. Among them the Haralick model made a great success [29]. Some researcher developed the traditional Haralick model from 2 dimensional (2D) to 3 dimensional (3D) and adds more features [30]. Some research used the CMs instead of HF in the convolutional neural network (CNN) based model [31]. GLCM counts the frequency of voxel-pairs of certain gray levels in the 2D/3D space and is defined by two important parameters, i.e., direction and displacement as shown in Fig. 4. In 2D case, there are 8 sampling directions considering first neighboring pixels (Fig. 4a), while there are 26 different directions in volumetric space (Fig. 4b). Every direction would create one GLCM. Since GLCM is symmetry invariant, we only need to calculate in one direction without in its inverse direction. Therefore, we calculated 13 GLCMs in 3D space, where the calculating procedure was illustrated in Fig. 4c.

**Fig. 5:**
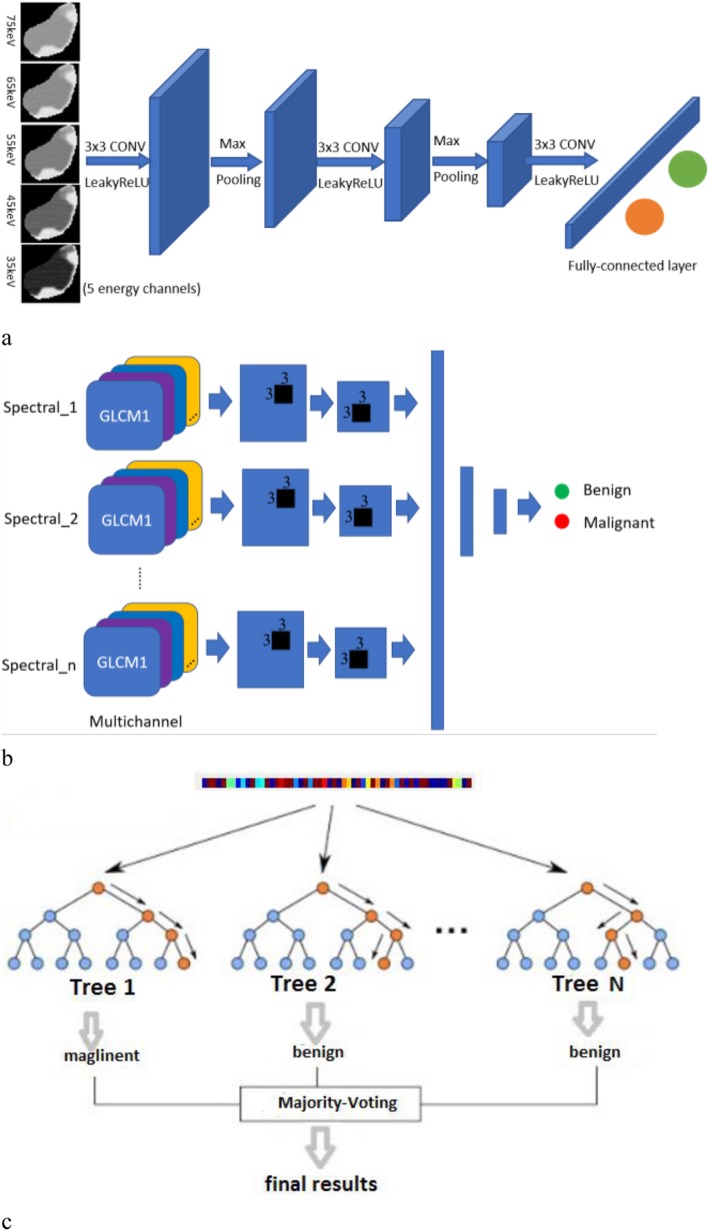
Classification models for three different inputs: (**a**) the computed tomography raw image-based convolutional neural network (CNN) model, (**b**) the co-occurrence matrice based CNN model and (**c**) the Haralick feature based random forest model

This paper investigated these three type inputs, i.e., CT images, CMs and HF to validate the effectiveness of the spectral enhanced texture and explore possible ways for the classification tasks. An example of the CMs and HF texture features is also presented in Fig. 3 (2nd and 3rd rows). It is noted the CMs and HF has 13 sampling directions. We only demonstrate one direction as an example. We could see both the texture features in HF and CM also vary prominently among different energies. For the three type inputs, three models were implemented: the CT raw image-based CNN model (IM-CNN), the CM based CNN model (CM-CNN) and the HF based random forest model (HF-RF). The three models are illustrated in the Fig. 5. Details of each model are presented in the following.

IM-CNN: The slice with largest cross-section was chosen as the input. A multi-channel network structure was designed, which takes each energy 2D image as one input channel and multiple channels are used to take all energy images from spectral CT. A five-channel design was shown in Fig. 5a. The model consists of seven layers including three convolution layers, two max-pooling layers and two fully connected layers. In each convolution layer, batch normalization and activation function are performed. It uses the rectified linear unit (ReLU) as the activation function, the cross-entropy loss as the training loss and softmax function at the last fully connected layer.

CM-CNN: Texture-based Gray-level Co-occurrence Matrix (GLCM) was used as inputs for this model. A multi-channel network structure was designed, which takes each energy 2D GLCM as one input channel and multiple channels are used to take all energy GLCM from spectral CT. The multiple-channels design (n channels as an illustration) was shown in Fig. 5b. The model consists of seven major layers including two convolution layers, two max-pooling layers and three fully connected layers. In each convolution layer, batch normalization and activation function are performed. It uses the ReLU as the activation function, the cross-entropy loss as the training loss and softmax function at the last fully connected layer.

HF-RF: HF as a typical texture descriptor are extracted from GLCM, which is also handcrafted texture features defined in ref. [29]. According to ref. [29], every GLCM could deduce 14 Haralick measures. Twenty-eight HF would be generated by calculating means and ranges of Haralick measures over 13 directions. Then the texture descriptor consisting of 28 HFs is fed to random forest (RF) classifier to perform classification [26]. For each process of RF, the descriptors of all polyps are divided into training groups and testing groups. Before classification, we first calculate the priority of each variable in texture descriptor. Gini coefficient is introduced to be the priority measurement. Next, some variable sets are generated using the forward step feature selection method on the ranked variables. This architecture was shown in Fig. 5c. Classifications are performed under the parameters of 3000 trees and $\sqrt{28}$ randomly selected variables for each node.

### Evaluation strategies

Currently, the photon counting spectral CT is under research and development and has not been utilized in clinical. Therefore, the evaluation was performed on simulated spectral CT datasets.

### Image reconstruction evaluation

VMI obtained from the dual energy CT can be used to mimic the output of the PCCT. The procedure can be described as follows. Due to different materials have different spectral attenuation response, the Dual energy CT will generate VMI based on two measurements of different energy. Then the photon counts for each energy channel can be calculated using the forward projection. Poisson noise is superimposed to the signal to consider the statistical property. Then the low counts projection was reconstructed by the proposed LRTP method.

### Classification evaluation

**Fig. 6:**
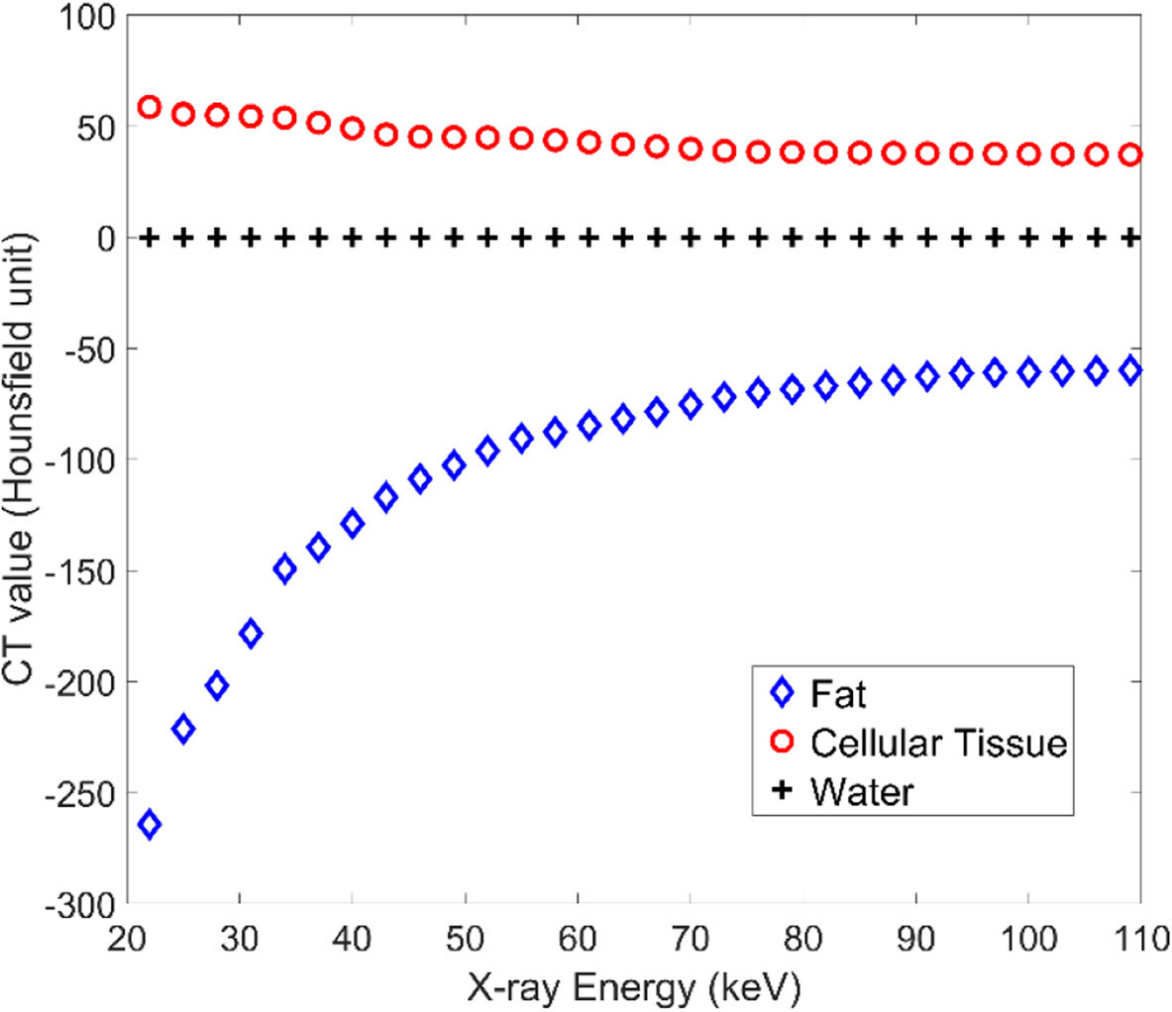
Computed tomography values of three tissue types under different energy

The classification models should be evaluated on the datasets with pathology reports as the ground truth. Therefore, the evaluation was performed on our pathology proven colon polyp datasets. However, we do not have VMI images for this dataset. As a way around, we simulated the spectral CT data based on attenuation physical model. Colon polyps consist of soft tissue, which can be represented by three tissue types, i.e. fat, cellular tissue and water. Each tissue type has different attenuation coefficient or CT value at different X-ray energy. The CT-energy response curve of the three tissue types [32] to the X-ray energy in Hounsfield units is shown in Fig. 6. Water is used as the reference material of which the CT value is zero. The cellular tissue has positive values, while the CT values for fat tissue are always negative. Once we have the CT data at one energy, we can use a linear scaling method to simulate the CT data at other energies according to the response curve for each tissue type.

A two-fold cross-validation method was used to evaluate the performance via the mean and the standard deviation of area under the receiver operating characteristic curve (AUC). We compared the performance of all three models at each individual X-ray energy and the combination of five energy channels. We also compared the performance of all three models based on the difference images. The difference images are obtained by subtracting the CT data of the neighboring energy channels. Then we extracted the CMs and HF features based on the difference images. More details will be described in experiments and results section.

## Experiments and results

### Dataset

Dataset 1 A patient who revealed suspected pulmonary tumor appearing as nodules was scanned using a dual-energy high-definition CT scanner (GE Discovery CT750 HD) of the chest in gemstone spectral imaging mode. The VMI at 80 and 140 kVp with the thickness of 2.5 mm and the spacing of 0.75 mm were transferred to an AW 4.4 workstation for analysis. The VMI were generated at 10 keV monochromatic energy level increments from 60 to 100 keV, resulting in one set of 5 images, which represents the VMI for each channel. A representative slice was specified from the image volume to evaluate our methodology LRTP for PCCT image reconstruction

Dataset 2 Fifty-nine patients with 63 polyps, including 31 benign and 32 malignant, were scanned in conventional CT facility with effective 75 keV X-ray energy. Each abdominal CT image volume consists of more than 400 image slices. All polyps were resected with following pathology reports to verify whether each polyp was malignant as an adenocarcinoma or benign as an adenomatous. Summary of the polyp mass with its pathology results are presented in Table 2. Based on the CT-energy response curve in Fig. 6, those 63 volumetric polyp CT images were used to simulate the CT data in Hounsfield units at other effective energies from 35 keV to 65 keV

**Table 2:** Summary of ployp mass for dataset 2

Category	Pathology	Count	Male: Female	Average size (mm)
Benign (0)	Serrated adenoma	3	2:1	34.3
	Tubular adenoma	2	2:0	35.0
	Tubulovillous adenoma	21	11:10	37.6
	Villous adenoma	5	4:1	55
Malignant (1)	Adenocarcinoma	32	12:20	43.9

### Results of image reconstruction

The dataset 1 was used to evaluate the texture enhanced image reconstruction algorithm, LRTP. For comparison study, some well-established methods were employed, including the simultaneous algebraic reconstruction technique (SART), total variation minimization (TV) [33], low-rank representation and total variation regularization (LRTV) [34] as well as tensor dictionary learning (TDL) [35].

**Fig. 7:**
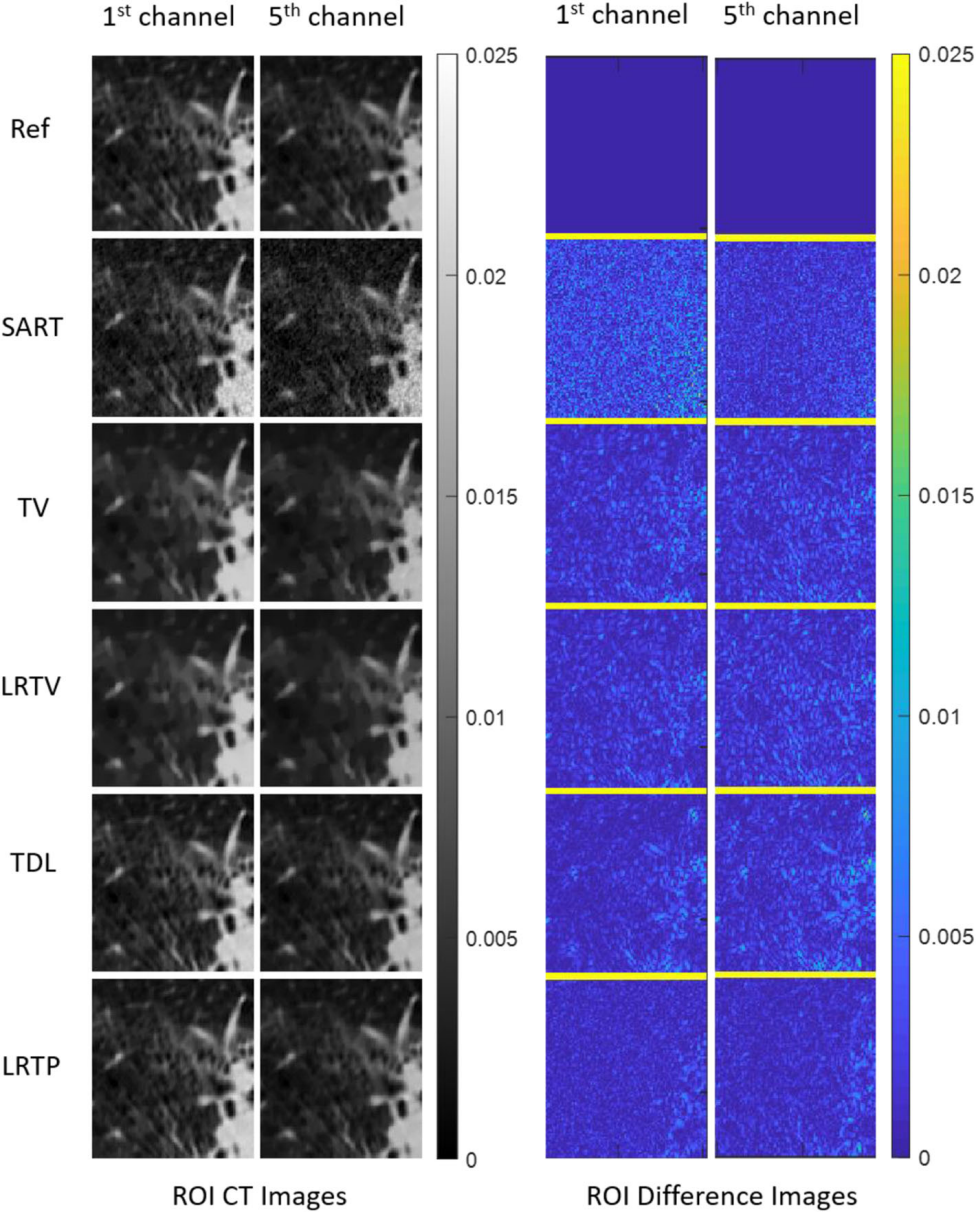
Close-up views for comparison of lung region of interests reconstructed by different algorithms. The gray level images are the magnified lung ROI CT images in the red box in Fig. 1. The corresponding absolute difference images are shown in color. From top to bottom are the reference images reconstructed from full-dose projections by SART and the low-dose images reconstructed by SART, TV, LRTV, TDL and LRTP methods, respectively. *ROI* Region of interest; *CT* Computed tomography; *TV* Total variation; * LRTV* Low rand total variation; *TDL*Tensor dictionary learning; *SART* Simultaneous algebraic reconstruction technique; *LRTP* Low rank texture prior

The reconstructed images are zoomed in the lung region and shown in Fig. 7. The gray images are the magnified lung ROI CT images. The corresponding absolute difference images are shown in color. From top to bottom are the reference images reconstructed from full-dose projections by SART and the low-dose images reconstructed by SART, TV, LRTV, TDL and LRTP methods. The SART reconstructed images contain strong noise artifacts and some texture features are drowned out by noise. The strong random noise can be clearly seen in the absolute difference images. TV and LRTV methods significantly reduced the noise but most of the small texture features, such as the blood vessels were smoothened. The corrupted texture can be clearly observed in their absolute difference images. TDL method could identify these texture features, and the details were retained without blurring. The proposed LRTP method yielded very clear images and the smallest difference with the reference image comparing with other methods.

Quantitative measurements are shown in Table 3. The proposed LRTP had the lowest root mean square error (RMSE) and the highest peak signal to noise ratio for all energy channels. The structure similarity index (SSIM) and feature similarity index (FSIM) also employed to measure the structure similarity and feature similarity between the references and reconstructed images. In Table 3, the proposed LRTP method obtain the greatest SSIM and FSIM values than other competing methods in all channels which further demonstrates that the LRTP method has the best image quality in terms of quantitative assessment.

**Table 3:** RMSE (10e-4), PSNR, SSIM, FSIM Index for region of interest images in Fig. 7

Metrics	Methods	Reconstructed images (channel number)				
		1th	2th	3th	4th	5th
RMSE	SART	6.5373	72,168	9.1134	9.8020	10.6390
	TV	6.3068	5.7223	5.9562	6.0101	5.9182
	LRTV	6.4144	5.5096	5.5894	5.4789	5.3151
	TDL	6.7663	5.4743	5.4770	5.2326	4.9444
	LRTP	4.7431	3.8528	3.8517	4.0398	4.2506
PSNR	SART	30.624	29.054	27.027	26.270	25.401
	TV	30.922	31.069	30.721	30.518	30.495
	LRTV	30.775	31.398	31.273	31.322	31.429
	TDL	30.311	31.453	31.449	31.721	32.057
	LRTP	33.397	34.505	34.507	33.969	33.370
SSIM	SART	0.9994	0.9993	0.9989	0.9987	0.9984
	TV	0.9992	0.9993	0.9993	0.9993	0.9993
	LRTV	0.9992	0.9993	0.9993	0.9994	0.9994
	TDL	0.9992	0.9994	0.9994	0.9995	0.9995
	LRTP	0.9995	0.9997	0.9997	0.9997	0.9997
FSIM	SART	0.9981	0.9975	0.9958	0.9947	0.9920
	TV	0.9950	0.9941	0.9942	0.9950	0.9952
	LRTV	0.9944	0.9943	0.9942	0.9948	0.9952
	TDL	0.9963	0.9970	0.9970	0.9973	0.9975
	LRTP	0.9988	0.9992	0.9992	0.9991	0.9989

*TV* Total variation, *LRTV* Low rand total variation, *TDL* Tensor dictionary learning, *SART* Simultaneous algebraic reconstruction technique, *LRTP* Low rank texture prior, *RMSE* Root of mean square error, *PSNR* Peak signal to noise ratio, *SSIM* Structure similarity index, *FSIM* Feature similarity index

### Results of classification

We explored three machine learning models in terms of three type inputs, i.e., raw CT images, texture images (GLCMs images), hand crafted features from texture images. The three type inputs described above were fed to the three models presented in Fig. 5. For each model, each individual energy channel data was used then the combination data of five-energy channels was used. For each experiment, we randomly split the dataset into two folds for 100 runs. At each run, we used one-fold data for training and the other for testing. Then we swapped the training and testing data. In the end, we obtained the averaged AUC for the 100 runs and its standard deviation. The results are shown in Table 4.

**Table 4:** Classification performance of raw image scheme

Energy (kev)	AUC (mean ± std)		
	CT image (single slice)	Co-occurrence matrix	Haralick features
75	0.585 ± 0.073	0.902 ± 0.061	0.887 ± 0.048
65	0.597 ± 0.069	0.899 ± 0.064	0.879 ± 0.050
55	0.606 ± 0.064	0.872 ± 0.070	0.909 ± 0.037
45	0.629 ± 0.055	0.867 ± 0.070	0.901 ± 0.044
35	0.631 ± 0.071	0.820 ± 0.086	0.913 ± 0.046
All	0.659 ± 0.069	0.906 ± 0.057	0.917 ± 0.039

*AUC* Area under the receiver operating characteristic curve, *CT* Computed tomography

For all three inputs, the five-energy spectral CT data gives the best performance. An improvement of the AUC score is obtained by 7.3%, 0.42% and 3.0% for the spectral images, CMs and HFs respectively on the five-energy spectral data over the original 75 kev data only. Comparing results among three inputs, we observed that GLCM feature image could provide more effective information than the CT image for the CNN learning. This agrees with our expectation that for the limited datasets with pathological ground truth, CMs based learning is more efficient than CT image-based learning since the GLCM is extracted from the raw image as an effective texture descriptor to reflect the lesion heterogeneity.

One alternative way to utilize the energy information is to use difference images. We obtain difference images by subtracting the CT data of the neighboring energy channels. Such we obtained four sets of difference CT images from the five energy channels. Similarly, we extract the CMs and HF from four difference CT images and fed the three type inputs into the corresponding models. The results of the difference image scheme are summarized in Table 5. Similar with raw image scheme, the five-energy spectral CT data gives the best performance. For individual difference images, the performance from all energy pairs are almost the same and better than the performance of corresponding raw image scheme. The CM-CNN model HF-RF and model achieves the best AUC of 0.934 and 0.927. Similarly, CMs and HF performed better than CT image, which have been discussed above.

**Table 5:** Results of the difference image scheme

Energy	AUC (mean ± std)		
	CT image (single slice)	Co-occurrence matrix	Haralick features
65–75	0.661 ± 0.061	0.923 ± 0.043	0.907 ± 0.040
55–65	0.656 ± 0.063	0.927 ± 0.037	0.914 ± 0.045
45–55	0.661 ± 0.062	0.918 ± 0.035	0.920 ± 0.042
35–45	0.671 ± 0.064	0.925 ± 0.034	0.914 ± 0.042
All	0.687 ± 0.068	0.934 ± 0.034	0.927 ± 0.044

## Discussion

In this paper, the proposed LRTP reconstruction method combines low-rank representation and TP under Bayesian framework. This combination considers the correlation among different channels while preserve the texture information in the specific tissue regions. Comparing the LRTP with LRTV (Fig. 7), we can clearly observe the tissue texture was much better enhanced by the proposed method. While, the similarity of TP among different energy channels demonstrates the LR property of the spectral CT data, how to future enhance the tissue texture using the TP similarity is one of our future research interests.

In the computer aided diagnosis (CADx), tissue texture plays an important role. The tissue texture can be enhanced by the spectral CT because each tissue always has different attenuation coefficient under different X-ray energy. In spectral CT, multiple datasets are obtained at multiple monotonic energy X-ray energies. By compiling all energy channel CT data, the tissue texture can be better represented and benefit the CADx. Using PCCT enhanced texture for clinical task has not been studied thoroughly as mentioned in introduction section. This paper is serving as pioneering work to explore how to utilize PCCT effectively especially given a limited dataset, which is always true in medical imaging field. We investigated three type inputs for the classification task. The overall tendency remains the same across three type inputs that the spectral CT model outperforms single energy data or traditional CT data. This validates the effectiveness of enriched texture by spectral CT. Moreover, the CMs and HF performs much better than the raw CT images, which shows that incorporating our knowledge into the model can help to improve the performance, especially in the limited datasets.

For reconstruction evaluation, we used the VMI from dual energy to mimic the signal of photon counting. For classification evaluation, we used the basic physics model to simulate the spectral CT data. This simulation assumes that each voxel is pure material, which means no partial volume effect was considered. Studies on real spectral CT data is another of our future research interests.

## Conclusions

Spectral CT is believed to be able to enhance the tissue contrast and enrich tissue texture information. In this study, we addressed two texture related issues for the spectral CT practical usage. One is to enhance the tissue texture of spectral CT images by the proposed LRTP algorithm. The other is to make use of the enriched texture for clinical tasks by investigating three type input models. All three models showed improved AUC results on the five-energy spectral CT data over the original single CT data only. The outcome also demonstrated the potential of PCCT in polyp classification tasks in the future clinical practice. Our innovation lies in two manifolds: (1) LRTP algorithm for PCCT reconstruction, which integrates the inter-channel correlation and the inner-channel spatial texture into a unified Bayesian reconstruction framework; (2) pioneering exploration of effective learning model using PCCT data for lesion classification task. This paper shows our effort to advance the potential of PCCT in practical usage by considering the upstream reconstruction and downstream classification model. The comprehensive texture themed study shows the insight of maximizing the application of clinical important prior information, e.g. tissue texture in this paper, into practical usage for performance improvement.

## Data Availability

The datasets used or analyzed during current study are private dataset.

## Abbreviations

2D: 2 dimensional
3D: 3 dimensional
AI: Artificial intelligence
AUC: Area under the receiver operating characteristic curve
CADx: Computer aided diagnosis
CM-CNN: CMs based CNN model
CMs: Co-occurrence matrices
CNN: Convolutional neural network
CT: Computed tomography
FDCT: Full dose CT
FSIM: Feature similarity index
GLCM: Grey level CMs
HF: Haralick features
HF-RF: HF based random forest model
IM-CNN: Image based CNN model
LRTP: Low rank texture prior
LRTV: Low rand total variation
MRF: Markov random field
PCCT: Photon counting CT
PSNR: Peak signal to noise ratio
RMSE: Root of mean square error
SART: Algebraic reconstruction technique
SSIM: Structure similarity index
TDL: Tensor dictionary learning
TP: Texture prior
TV: Total variation
VMI: Virtual mono-energetic image

## References

[1] Gatenby RA, Grove O, Gillies RJ (2013). Quantitative imaging in cancer evolution and ecology. Radiology.

[2] Bashir U, Siddique MM, Mclean E, Goh V, Cook GJ (2016). Imaging heterogeneity in lung cancer: techniques, applications, and challenges. AJR Am J Roentgenol.

[3] Ng F, Ganeshan B, Kozarski R, Miles KA, Goh V (2013). Assessment of primary colorectal cancer heterogeneity by using whole-tumor texture analysis: contrast-enhanced CT texture as a biomarker of 5-year survival. Radiology.

[4] Aerts HJWL, Velazquez ER, Leijenaar RTH, Parmar C, Grossmann P, Carvalho S (2014). Decoding tumour phenotype by noninvasive imaging using a quantitative radiomics approach. Nat Commun.

[5] Taguchi K, Iwanczyk JS (2013). Vision 20/20: single photon counting x-ray detectors in medical imaging. Med Phys.

[6] Kim K, Ye JC, Worstell W, Ouyang JS, Rakvongthai Y, El Fakhri G (2015). Sparse-view spectral CT reconstruction using spectral patch-based low-rank penalty. IEEE Trans Med Imaging.

[7] Niu SZ, Yu GH, Ma JH, Wang J (2018). Nonlocal low-rank and sparse matrix decomposition for spectral CT reconstruction. Inverse Probl.

[8] Li TF, Li X, Xing YX, Lu HB, Hsieh J, Liang ZR (2003) A strategy for reduction of streak artifacts in low-dose CT. In: abstracts of 2003 IEEE nuclear science symposium. Conference record, IEEE, Portland, OR, USA, 19-25 October 2003

[9] Chen Y, Gao DZ, Nie C, Luo LM, Chen WF, Yin XD (2009). Bayesian statistical reconstruction for low-dose X-ray computed tomography using an adaptive-weighting nonlocal prior. Comput Med Imaging Graph.

[10] Zhang H, Han H, Wang J, Ma J, Liu Y, Moore W (2014). Deriving adaptive MRF coefficients from previous normal-dose CT scan for low-dose image reconstruction via penalized weighted least-squares minimization. Med Phys.

[11] Zhang H, Han H, Liang ZR, Hu YF, Liu Y, Moore W (2016). Extracting information from previous full-dose CT scan for knowledge-based Bayesian reconstruction of current low-dose CT images. IEEE Trans Med Imaging.

[12] Gao YF, Liang ZR, Moore WH, Zhang H, Pomeroy MJ, Ferretti JA (2019). A feasibility study of extracting tissue textures from a previous full-dose CT database as prior knowledge for Bayesian reconstruction of current low-dose CT images. IEEE Trans Med Imaging.

[13] Gao YF, Tan JX, Shi YY, Lu SM, Liang ZR (2019) A machine learning approach to construct a tissue-specific texture prior from previous full-dose CT for Bayesian reconstruction of current ultralow-dose CT images. In: abstracts of the 15th international meeting on fully three-dimensional image reconstruction in radiology and nuclear medicine, SPIE, Philadelphia, United States, 28 may 2019. 10.1117/12.2534441

[14] Shi Y, Gao Y, Liang Z (2019) Spectral CT reconstruction via low-rank representation and tissue-specific texture preserving markov random field regularization. In: abstracts of IEEE nuclear science symposium and medical imaging conference, Manchester central convention Centre, Manchester, 26 October-2 November 2019

[15] Matsumoto K, Jinzaki M, Tanami Y, Ueno A, Yamada M, Kuribayashi S (2011). Virtual monochromatic spectral imaging with fast kilovoltage switching: improved image quality as compared with that obtained with conventional 120-kVp CT. Radiology.

[16] Lv PJ, Lin XZ, Li JY, Li WX, Chen KM (2011). Differentiation of small hepatic hemangioma from small hepatocellular carcinoma: recently introduced spectral CT method. Radiology.

[17] Lv PJ, Lin XZ, Gao JB, Chen KM (2012). Spectral CT: preliminary studies in the liver cirrhosis. Korean J Radiol.

[18] Pan ZL, Pang LF, Ding B, Yan C, Zhang H, Du LJ (2013). Gastric cancer staging with dual energy spectral CT imaging. PLoS One.

[19] Al Ajmi E, Forghani B, Reinhold C, Bayat M, Forghani R (2018). Spectral multi-energy CT texture analysis with machine learning for tissue classification: an investigation using classification of benign parotid tumours as a testing paradigm. Eur Radiol.

[20] Setio AAA, Ciompi F, Litjens G, Gerke P, Jacobs C, Van Riel SJ (2016). Pulmonary nodule detection in CT images: false positive reduction using multi-view convolutional networks. IEEE Trans Med Imaging.

[21] Jiang HY, Ma H, Qian W, Gao MD, Li Y (2018). An automatic detection system of lung nodule based on multigroup patch-based deep learning network. IEEE J Biomed Health Inform.

[22] Wang HF, Zhao TT, Li LC, Pan HX, Liu WQ, Gao HQ (2018). A hybrid CNN feature model for pulmonary nodule malignancy risk differentiation. J X-Ray Sci Technol.

[23] Tan JX, Huo YM, Liang ZR, Li LH (2019). Expert knowledge-infused deep learning for automatic lung nodule detection. J X-Ray Sci Technol.

[24] Han H, Li LH, Han FF, Song BW, Moore W, Liang ZR (2015). Fast and adaptive detection of pulmonary nodules in thoracic CT images using a hierarchical vector quantization scheme. IEEE J Biomed Health Inform.

[25] Andersen AH (1989). Algebraic reconstruction in CT from limited views. IEEE Trans Med Imaging.

[26] Liu L, Fieguth PW (2012). Texture classification from random features. IEEE Trans Pattern Anal Mach Intell.

[27] Hong XP, Zhao GY, Pietikäinen M, Chen XL (2014). Combining LBP difference and feature correlation for texture description. IEEE Trans Image Process.

[28] Xia GS, Liu G, Bai X, Zhang LP (2017). Texture characterization using shape co-occurrence patterns. IEEE Trans Image Process.

[29] Haralick RM, Shanmugam K, Dinstein I (1973). Textural features for image classification. IEEE Trans Syst, Man, Cybern.

[30] Hu YF, Liang ZR, Song BW, Han H, Pickhardt PJ, Zhu W (2016). Texture feature extraction and analysis for polyp differentiation via computed tomography colonography. IEEE Trans Med Imaging.

[31] Tan JX, Gao YF, Cao WG, Pomeroy M, Zhang S, Huo YM, et al (2019) GLCM-CNN: gray level co-occurrence matrix based CNN model for polyp diagnosis. In: Abstracts of 2019 IEEE EMBS international conference on Biomedical & Health Informatics, IEEE, Chicago, IL, USA, 19-22 may 2019. DOI: 10.1109/BHI.2019.8834585

[32] NIST Standard Reference Database 126 (2004) Physical Measurement Laboratory, Gaithersburg. https://www.nist.gov/pml/x-ray-mass-attenuation-coefficients.

[33] Gong CF, Han C, Gan GH, Deng ZX, Zhou YQ, Yi JL (2017). Low-dose dynamic myocardial perfusion CT image reconstruction using pre-contrast normal-dose CT scan induced structure tensor total variation regularization. Phys Med Biol.

[34] Chu JY, Cong WX, Li L, Wang G (2013). Combination of current-integrating/photon-counting detector modules for spectral CT. Phys Med Biol.

[35] Zhang YB, Mou XQ, Wang G, Yu HY (2016). Tensor-based dictionary learning for spectral CT reconstruction. IEEE Trans Med Imaging.

